# Case Series of Patients with Ruptured Abdominal Aortic Aneurysm

**DOI:** 10.5811/westjem.2015.3.24027

**Published:** 2015-04-06

**Authors:** Taylor Spencer, Rushad Juyia, Robyn Parks, Matthew Hodapp

**Affiliations:** Albany Medical Center, Department of Emergency Medicine, Albany, New York. West Virginia University Health Center, Morgantown, West Virginia

## Abstract

**Introduction:**

Traditionally, patients with suspected ruptured abdominal aortic aneurysm (rAAA) are taken immediately for operative repair. Computed tomography (CT) has been considered contraindicated. However, with the emergence of endovascular repair, this approach to suspected rAAA could be changing.

**Methods:**

We present retrospective data in a case series of 110 patients with rAAA. Patients were managed at a single tertiary medical center over a five-year period. At this site, there was an established multidisciplinary protocol in which patients with suspected rAAA undergo CT with consideration for endovascular aortic repair (EVAR).

**Results:**

Our results demonstrated a mortality of 30% with our institutional protocol for CT in suspected rAAA. Comparing patients who ultimately had EVAR with open repair, those able to have endovascular aneurysm repair (EVAR) had lower mortality, shorter hospital stays for survivors, and a greater likelihood of being discharged to home than those with open repair. While survivors were more likely to have had EVAR, surviving patients were younger, had a significantly lower creatinine at presentation, and required fewer blood transfusions than those who died.

**Conclusion:**

Based on this case series, an institutional approach endorsing CT for presumed rAAA appears to be reasonable. Our results suggest that EVAR may be beneficial in appropriately-selected patients and that CT may potentially facilitate superior management options for patient care.

## INTRODUCTION

The traditional dogma is explicit regarding the management of probable ruptured abdominal aortic aneurysm (rAAA). A patient presumed to have rAAA should be taken for immediate surgical repair, rather than undergo confirmatory computed tomography (CT).^1^ Tintinalli notes that, even when reaching the operating room, half of patients with rAAA die, and therefore argues “imaging modalities should be restricted to patients who are considered unlikely to have a ruptured AAA.”^2^

However, in the age of endovascular repair, traditional dogma may be crumbling. CT may facilitate endovascular aortic repair (EVAR), and emergent unstable patients with rAAA are increasingly considered candidates for EVAR.^3^–^5^ EVAR is associated with lower mortality, shorter hospital stay, and greater likelihood of discharge to home, although patient selection complicates these conclusions.^3^,^5^–^10^

In light of the emerging role of EVAR in rAAA – and in turn, the role for CT in presumed rAAA – we present preliminary data from a single-center experience with deviation from this established dogma.

## METHODS

This brief report presents a case series of patients with ruptured abdominal aortic aneurysm from September 2005 to November 2010. These patients were managed by the vascular surgery group at a large tertiary medical center where CT with contrast was considered standard care prior to surgical intervention as part of a multidisciplinary protocol initiated in 2002. The Figure below illustrates the Albany Medical Center protocol for rAAA.

Consistent with Worster and Bledsoe’s summary of proper methods for retrospective chart review,^11^ we obtained the patient list from an existing database of vascular surgery patients. Selection criteria included all inpatients admitted through emergency department (ED) with a diagnosis of rAAA from billing records, regardless of whether surgical intervention was ultimately pursued. Our sample was all patients who met the inclusion criteria. Abstractors were trained prior to data collection, with subsequent performance monitoring. Two medical students blinded to the study’s purpose extracted data from medical records. A standardized data extraction form was used, with variables defined in advance and a standardized sequence for identifying data. This study was approved by the local institutional review committee.

We performed data analysis with a combination of Excel and stata. Demographic statistics were calculated median and IQRs. For comparison between groups on nominal data, we used either chi-squared or Fischer exact test, as appropriate. Then an F-test was first used to determine equality of variances, followed by unpaired, two tailed Student’s t-tests to compare subsets of patients.

## RESULTS

The case series included 110 patients with a median age of 74 years (IQR = 65–81 years). The age range was 39–95 years. It included 35 females and 75 males. The median measured AAA size in largest dimension was 7.75cm (IQR=6.45–9.4cm). Of the subjects, 82 had no prior aortic repair, 21 had prior aortic repair, and 7 were not recorded. Out of 105 patients with a recorded blood pressure, 40 were hypotensive (38.1%), defined as less than 90/60mmHg. Additionally, of 102 patients with a recorded heart rate, 30 were tachycardic (29.4%). Defining acute shock by hypotension or tachycardia, 60 out of the 106 patients that had a blood pressure or heart rate recorded were in shock (56.6% of patients). Median intensive care unit (ICU) length of stay was four days (IQR=1–8days), with 18 patients having zero ICU days reported and a range from 0–46 days. Median hospital length of stay was 10 days (IQR=3–19days), including ten with zero days (early deaths) and a range from 0–92 days. The ultimate outcome included 33 total deaths, for a mortality rate of 30%. Of the 77 survivors, 39 were discharged to home, 7 were discharged with home health services, 22 were discharged to rehab, 8 were discharged to a skilled nursing facility, and one was transferred to the Veterans Affairs health system.

In this case series, 57 patients had EVAR and 48 patients had open repair. The open repairs included three patients who started with EVAR and necessitated conversion to open, or 5% of the cases planned as EVAR. Those who underwent EVAR had smaller aneurysms compared to the open repair group. In the open repair cohort, significantly more units of packed red blood cells (pRBCs) were transfused (p=0.018), and creatinine rose significantly higher (p=0.019) as noted in Table 1. EVAR patients had appreciably lower mortality rates than open repair (p=0.028).

We then compared the 33 patients with rAAA who died with 77 survivors, as seen in Table 2. The rates of prior repair were comparable for those who lived and those who died (p=0.779), but those who died were significantly older (p<0.001), had higher initial creatinine (p=0.02), and required almost twice as many units of pRBCs as survivors (p=0.002). Those who died trended towards a higher peak creatinine (p=0.06). There were no differences between the two groups in relation to sex, highest heart rate, or lowest systolic blood pressure (p=0.503, p=0.375, p=0.378, respectively). Mortality did not correlate with AAA size (p=0.582). Open repair patients had appreciably higher mortality rates than EVAR in a 2×2 contingency table (p=0.028).

Notably, of the 33 patients who died, nearly two thirds died on the day of presentation or the first full hospital day. The data suggest that this early mortality population skewed some results. It contributed to the shorter ICU and hospital stays, and to the greater transfusion needs in the group who died. In contrast, early mortality may have blunted the association of higher peak creatinine in those who died, which only reaches significance when the early mortality group is excluded (p<0.001). The majority of deaths are in this early mortality group.

There is much room for further study. More robust evidence might verify the benefits of EVAR and clarify when it is most appropriate, perhaps by randomized controlled trial (although likely to be unblinded). But this case series indicates that the traditional dogma should be questioned, and that sites may safely pursue pre-surgical CT in patients with potential ruptured aneurysms.

## DISCUSSION

This brief report describes a case series of patients with rAAA at a tertiary medical center where the standard approach is for CT, despite the traditional dogma. We summarize the patient population and outcomes. Notably, the mortality rate of 30% is less than the mortality reported by others, including 50% reported by Tintinalli.^2^,^3^,^12^

Patients with rAAA managed with EVAR had more favorable outcomes in this study. Our results are consistent with prior evidence that EVAR in emergency situations is associated with lower mortality, shorter hospital stays for survivors, and a greater likelihood of being discharged home.^3^,^7^,^9^,^12^ Presumably, our results bolster the argument that EVAR may be beneficial in appropriately-selected patients.

Aside from the association between EVAR and survival, our study demonstrated that patients who survived rAAA were younger, had a significantly lower creatinine at ED presentation, and required fewer pRBC transfusions. Mortality did not correlate with AAA size. Both ICU stay and the hospital length of stay were significantly longer for survivors, but this is skewed by the fact that a large number of mortalities occurred early.

Some have theorized an advantage of EVAR is the possibility of using local rather than general anesthesia.^10^,^13^–^15^ But in our case series, a survival advantage to EVAR persists even though all cases are performed under general anesthesia. Unlike open repair, EVAR avoids laparotomy and aortic cross clamping, both of which are associated with significant physiologic burden.^7^,^10^ This may account for our results, and the lower peak creatinine and reduced transfusion requirements might be markers of this.

## LIMITATIONS

Our brief report addresses the experience at a single center with CT preceding surgical interventions for rAAA. It may not be generalizable to sites without 24-hour radiology or vascular surgeons with significant experience with EVAR.

Demographics, lab values and other data were limited by the modest number of patients included. This could have underpowered certain analyses and underestimated the significance of some results. Despite this, we obtained noteworthy results.

In this nonrandomized observational case series, differences in survival between EVAR and open repair may be due to differences in the patient populations. Specifically, the nature of aneurysms necessitating open repair – rather than the actual operative intervention itself – may underlay some differences seen. Retrospective data can be complicated by potential limitations or ambiguities in the available records. Therefore, we considered all patients with rAAA, including transfers and those seen primarily at our site. We were not able to control for the degree of hemodynamic instability.

Furthermore, some of the findings were skewed because two thirds of the non-surviving patients died early (on the day of presentation or first full day of hospitalization). This early mortality group may have driven the associations between mortality and shorter lengths of stay or greater transfusion needs. But a correlation between higher peak creatinine and mortality emerges when this early mortality group is excluded. Such subgroup analysis should be interpreted with caution due to the small numbers.

## CONCLUSION

Traditional dogma is that patients with rAAA should not undergo CT imaging, but have immediate open surgery. However, in our institution, the standard is for CT imaging in the initial evaluation of rAAA.

From our preliminary case series, an institutional approach endorsing CT for presumed rAAA appears to be reasonable. This brief report demonstrates results at one site when routine CT is used. In this report, interpretation is not confounded by use of regional anesthesia in EVAR. The experience at our institution has been positive and encourages the use of preoperative CT in cases of rupture, even when patients may be unstable.

## Figures and Tables

**Figure:**
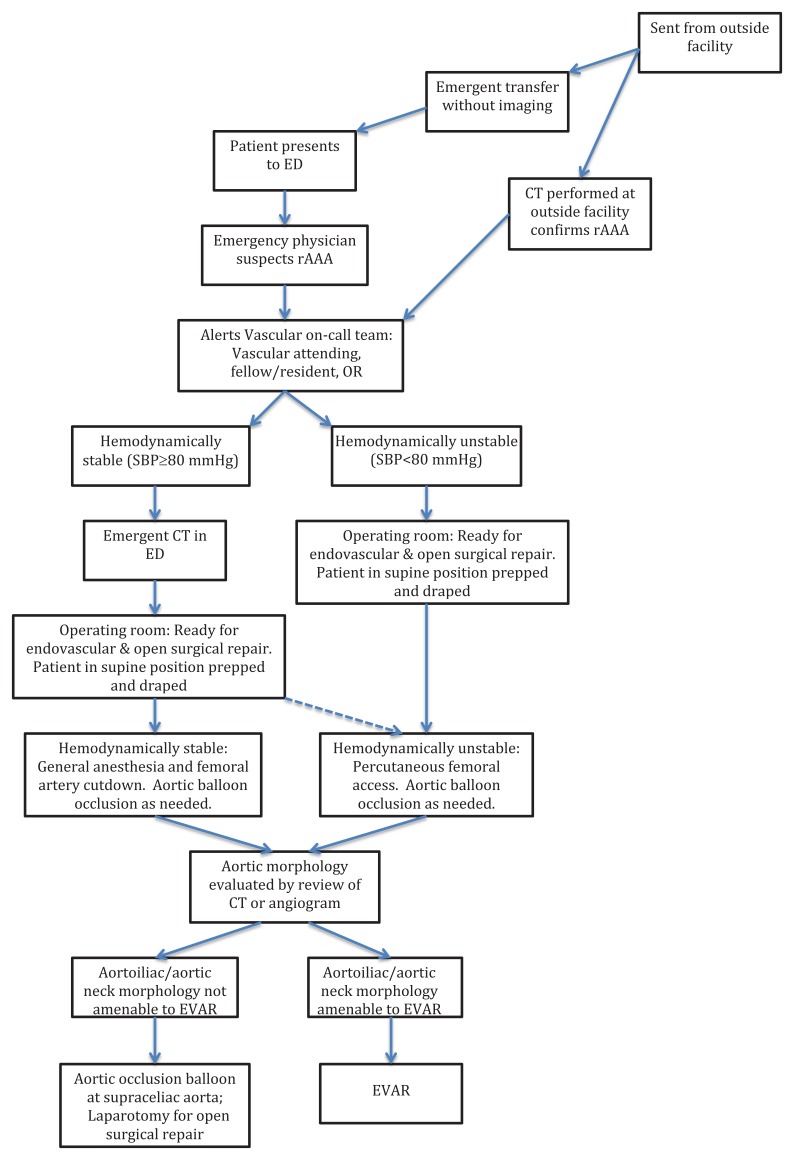
Albany Medical Center protocol for ruptured abdominal aortic aneurysm (rAAA). *ED,* emergency department; *CT,* computerized tomography, *OR,* operating room; *SBP,* systolic blood pressure; *EVAR,* endovascular aneurysm repair Albany Vascular Group standardized protocol for EVAR of ruptured rAAA. (Modified from Mehta M, Taggert J, Darling RC 3rd, et al. Establishing a protocol for endovascular treatment of ruptured abdominal aortic aneurysms: Outcomes of a prospective analysis. *J Vasc Surg*. 2006;44(1):1–8.).

**Table 1:** Comparison of endovascular and open repair.

	EVAR (IQR)	OPEN (IQR)	Significance
Aneurysm size (cm)	7 (5.5–8.3)	8.75 (7.2–10.3)	p=0.014
pRBCs transfused (units)	4 (1–8)	8 (5.5–12)	p=0.018
Peak creatinine (mg/dL)	1.3 (1–1.75)	2.2 (1.35–3.2)	p=0.019
Hospital days (survivors)	9 (4–16)	13.5 (3.5–23.5)	p=0.028
Mortality (%)	17.9	37.5	p=0.028

*EVAR,* endovascular aneurysm repair; *OPEN*, open repair; *pRBC*, packed red blood cells

**Table 2:** Comparison of patients with ruptured abdominal aortic aneurysm: survivors and mortality.

	Survivors (IQR)	Mortality (IQR)	Significance
Age (years)	71 (62–79)	78 (73–86.5)	p<0.001
Prior AAA Repair (%)	20.8	22.7	p=0.779
Entry creatinine (mg/dL)	1.2 (0.9–1.5)	1.55 (1.25–1.95)	p=0.024
ICU Days	5 (2–9)	1 (0–4.5)	p=0.042
Hospital days	12 (3–22)	1 (0–11.5)	p=0.009
pRBCs transfused (units)	4 (2–8)	8 (6–13.5)	p=0.002
Male (%)	70.1	63.6	p=0.503
Highest heart rate	88 (77–104.5)	87 (81–112)	p=0.375
Lowest systolic blood pressure	110 (91–127.5)	97 (70–128)	p=0.378
AAA size (cm)	7.55 (6.45–9.15)	8.2 (6.6–10.6)	p=0.582
Highest creatinine (mg/dL)	1.5 (1.1–2.6)	1.85 (1.3–3.7)	p=0.055

*AAA*, abdominal aortic aneurysm; *ICU*, intensive care unit; *pRBC*, packed red blood cells
